# Passive Immunoprophylaxis for the Protection of the Mother and Her Baby: Insights from In Vivo Models of Antibody Transport

**DOI:** 10.1155/2017/7373196

**Published:** 2017-01-11

**Authors:** Yanqun Xu, Iftekhar Mahmood, Lilin Zhong, Pei Zhang, Evi B. Struble

**Affiliations:** ^1^Division of Plasma Protein Therapeutics, Office of Tissues and Advanced Therapies, CBER/FDA, Plasma Derivatives Branch, Silver Spring, MD, USA; ^2^Division of Clinical Evaluation and Pharmacology/Toxicology, Office of Tissues and Advanced Therapies, CBER/FDA, Silver Spring, MD, USA

## Abstract

Pregnant women are at high risk for infection by pathogens. Vertical transmission of infectious agents, such as Zika, hepatitis B, and cytomegalovirus during pregnancy, remains a public health problem, associated with dire outcomes for the neonate. Thus, a safe prophylactic and therapeutic approach for protecting the mother and the neonate from infections remains a high priority. Our work is focused on better understanding the safety and efficacy determinants of IgG antibody preparations when used during pregnancy to benefit the mother and her baby. Using pregnant guinea pigs, we demonstrated that biodistribution of administered IgG to the fetus increases with gestation and results in lower maternal and higher fetal antibody concentrations as pregnancy progresses. Data suggests that partition of antibody immunotherapy to the fetal compartment may contribute to a lower maternal exposure (as measured by the AUC) and a shorter mean residence time of the IgG therapeutic at the end of pregnancy compared to nonpregnant age-matched controls, irrespective of the administered dose. Our studies provide insights on the importance of selecting an efficacious dose in pregnancy that takes into account IgG biodistribution to the fetus. The use of appropriate animal models of placental transfer and infectious disease during pregnancy would facilitate pharmacokinetic modeling to derive a starting dose in clinical trials.

## 1. Introduction

Infectious diseases are a significant contributor to pregnancy related maternal morbidity and mortality [1] accounting for more than 10% of pregnancy related deaths in the US [2]. Changes in immune status during pregnancy render women more susceptible to infections and, when infected, prone to more severe disease [3, 4]. Infections in pregnancy are associated with poor outcomes for the newborn, ranging from premature birth to congenital abnormalities and death [4–8]. Maternal immunity to pathogens improves outcomes; thus a significant emphasis has recently been placed on immunization of pregnant women in the US [9]. For vaccines that are contraindicated or not recommended during pregnancy, and for pathogens for which there are no approved vaccines, passive immunization with hyperimmune antibody preparations can be an alternative during pregnancy as there are no known risks to the fetus from such preparations [10]. However, in the few clinical studies where IgG was administered during pregnancy time-concentration data have often not been collected [11, 12]. Such information is critical, as the efficacy of IgG preparations has been shown to correlate with the dose [13] and the elevation of IgG trough levels is associated with reduced incidence of infections such as pneumonia [14].

Because intact IgG molecules can pass the placenta in a receptor-mediated fashion [15], passive immunization of the pregnant woman during pregnancy is believed to benefit not only the mother but also her baby [16] and it has been proposed or is being studied for CMV [12], HBV [17, 18], rubella [19], and other infections, with mixed results. Gaps remain in our knowledge of the efficacious dose, frequency of administration during pregnancy, and the determinants of protection in preventing mother-to-child transmission. In addition, not all IgG subclasses traverse the placenta at the same rate [20], and the magnitude of the clinical benefit may depend on the isotype of the neutralizing antibodies for a specific pathogen.

It is clear there is a need for more data, and, until such gaps are bridged, animal studies can inform decisions regarding starting dose and frequency of administration in clinical studies. In pregnant guinea pigs we have demonstrated that pharmacokinetic properties of IgG therapeutics administered to animals at the end of pregnancy differ from those in nonpregnant controls and that these changes may correlate with the transplacental transfer to the fetus which increases with gestation.

## 2. Materials and Methods

### 2.1. Animal Studies

All animal procedures were performed in accordance with protocols approved by the CBER Animal Care and Use Committee as previously described [21]. Briefly, Hartley Albino (Crl:HA) guinea pigs were purchased from commercial sources and mated to produce timed pregnancies. For the pharmacokinetic study, a total of ten pregnant guinea pigs (*n* = 5/group) on day 65 ± 2 of pregnancy were weighed and a polyclonal commercial human IgG purified from pooled plasma of healthy donors with high titers of antibodies against Hepatitis B, HepaGam® (Emergent Biosolutions, 549 IU/mL and 41 mg/mL) was administered intravenously at a dose 50 or 100 IU/kg (~3.5 or ~7 mg/kg). Dose was chosen to correspond with the approved dose for infants born to mothers testing positive for hepatitis B [22]. Maternal blood samples for pharmacokinetic (PK) study were collected at 10, 30, and 60 minutes and then every day until delivery. All pregnant guinea pigs gave birth 2–6 days after test article administration. An additional ten age-matched nonpregnant controls (*n* = 5/group) received the same IgG doses; blood samples for the PK study were collected 10, 30, and 60 minutes after administration and then daily for 5 days. Blood was stored overnight at 4°C to coagulate and then spun in a benchtop centrifuge at 1500 ×g for 5 minutes. Serum was collected, transferred into fresh tubes, and then frozen at −80°C for storage.

For the IgG trough levels at different gestation ages study, five groups of pregnant sows, one for each gestation age, *n* = 4–7/group, were used. On gestation days (GD) 22 ± 1 (*n* = 6), 30 ± 1 (*n* = 6), 40 ± 1 (*n* = 7), 50 ± 1 (*n* = 7), and 60 ± 1 (*n* = 4), approximately corresponding to the end of first trimester, middle and end of second trimester, and middle and end of the third trimester, the animals were weighed and HepaGam (Emergent Biosolutions, 549 IU/mL and 41 mg/mL) was administered intravenously at a dose 100 IU/kg (0.182 mL/kg or ~7 mg/kg). Five days after injection, blood samples were collected from all dams, five of the litters on GD45, and all the litters of GD55 and 65* via* cardio- or cordocentesis; whole fetuses were collected from all the remaining animals. Five days after injection was used as the sampling point for multiple reasons that have been addressed before [21] and included lack of anti-human antibody response. In addition, results from this and previous pharmacokinetic studies [23] indicated that five days following HepaGam administration in guinea pigs is approximately 1.5 times the half-life of human IgG in this species and thus can be considered equivalent to the time point when C_min_ or trough antibody levels are achieved during IGIV therapy.

Fetuses were carefully separated from the placenta, cleaned with cold PBS, weighed, flash-frozen individually, and then homogenized by placing 50% tissue : PBS w : v mixture on ice with an OMNI TH apparatus (Omni International, Kennesaw, GA). The mixture was centrifuged at 10,000 ×g for 10 minutes at 4°C and the supernate frozen at −80°C for storage until use. Human IgG and anti-HBsAg neutralizing activity in the serum and tissue homogenates were determined with a human IgG ELISA kit (Assaypro, St. Charles, MO) and ETI-AB-AUK PLUS (DiaSorin, Saluggia, Italy), respectively. IgG subclasses were measured with a human IgG subclasses kit (Cell Sciences, Newburyport, MA). All samples were measured in duplicates; data points out of data fitting range or with CV > 15% were excluded from analysis and repeated measurements taken, if possible. The kits did not cross-react with guinea pig serum or homogenates from controls that did not receive human IgG.

### 2.2. Data Transformation and Analysis

Absorbance values from ELISA were transformed into maternal and fetal human IgG concentration or anti-HBs international units by fitting them to an equation derived from a five-parameter fit of the standard curve (SoftMax Pro, Molecular Devices). The assumption was made that IgG was distributed equally in fetal tissue and serum, and no adjustment was made for the concentration measured in total body homogenates versus serum. Human IgG concentrations (*μ*g/mL) from all litter-mates were averaged to obtain a litter average; the fetal : maternal concentration ratios were calculated by dividing the litter averages by human IgG concentration from the respective dam. The litter was used as the unit for statistical analysis. One-way ANOVA with Bonferroni post hoc analysis was used to compare gestation dependent human IgG concentrations or fetal : maternal concentration ratios (GraphPad Software, San Diego, CA). Two-way ANOVA was used to compare the concentrations or fetal : maternal concentration ratios of IgG subclasses in different gestations; *p* values < 0.05 were considered significant.

Maternal and fetal human IgG concentrations were tested for correlation; Pearson two-tailed test was used to look for presence of a linear relationship; *p* value < 0.05 was considered significant.

### 2.3. Pharmacokinetic (PK) Analysis

PK parameters from serum concentration-time data in pregnant and nonpregnant guinea pigs were estimated by noncompartmental analysis. These PK parameters were estimated as follows.

Half-life was calculated by regression analysis on the terminal phase of concentration-time data according to

(1) $$\begin{array}{c} Half\text{-}life=\frac{0.693}{k}, \end{array}$$

where *k* is elimination rate constant

(2) $$\begin{array}{c} \text{Clearance}\;\;CL=\frac{Dose}{AUC}, \end{array}$$

where AUC is the area under the curve calculated by the trapezoidal rule

(3) $$\begin{array}{c} \text{Mean residence time MRT}=\frac{\text{AUMC}}{\text{AUC}}, \end{array}$$

where AUMC is the area under the moment curve calculated by the trapezoidal rule

(4) Volume  of  distribution  at  steady  state  Vss=CL×MRT.

Statistical differences in PK parameters for each group were analyzed using two-way ANOVA with dose and pregnancy status as variables (GraphPad Software, San Diego, CA). For any parameter, if dose was not a significant source of variance, individual values were pooled in two groups according to pregnancy status and reanalyzed using Student's* t*-test; *p* < 0.05 was considered significant.

## 3. Results and Discussion

We administered HepaGam, a commercial hepatitis B specific human IgG (HBIG) preparation, intravenously to pregnant guinea pigs at the end of gestation and nonpregnant controls and then measured the concentration-time dependence of the administered antibodies until the time of parturition (2–6 days after administration). The average concentrations and a summary of pharmacokinetic parameters for pregnant and nonpregnant age-matched controls are shown in supplemental Figure 1S in Supplementary Material available online at https://doi.org/10.1155/2017/7373196 and Table 1, respectively.

**Figure 1:**
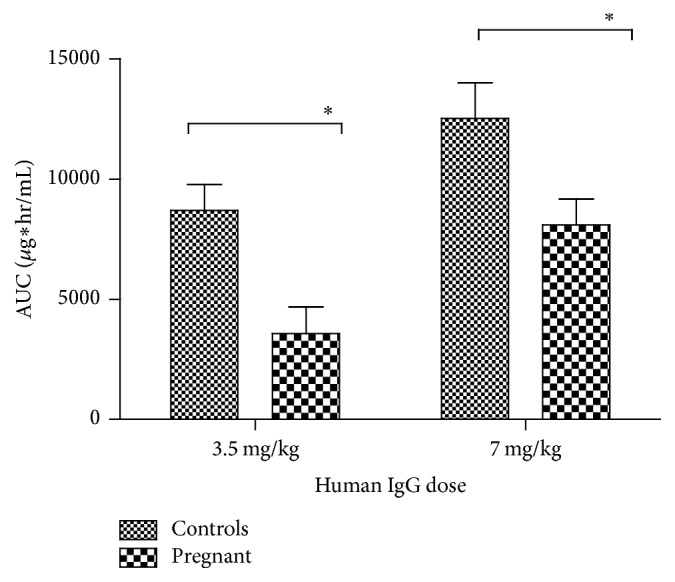
Area under the curve (AUC) of a hepatitis B immune globulin (HBIG) product in pregnant and nonpregnant guinea pigs. HBIG was administered intravenously at a dose 50 or 100 IU/kg (~3.5 and 7 mg/kg) in pregnant guinea pigs at the end of gestation (GD65 ± 2); age-matched nonpregnant animals served as controls. Human IgG exhibits lower AUC when it is administered at the end of pregnancy compared to nonpregnant animals, independent of dose; AUC shown as mean ± SEM, ^*∗*^*p* < 0.05 (Bonferroni post hoc analysis).

**Figure 2:**
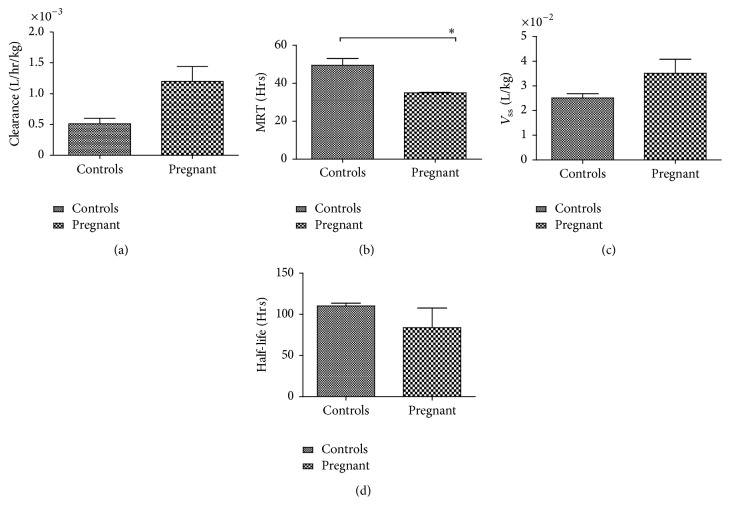
Pharmacokinetic (PK) parameters of a hepatitis B immune globulin (HBIG) product in pregnant and nonpregnant guinea pigs. Human IgG has larger clearance (a), lower mean residence time (MRT, (b)), larger volume of distribution at steady state (*V*_ss_, (c)), and lower half-life (d) when it is administered at the end of gestation (GD65 ± 2) compared to nonpregnant animals; data shown as mean** ± **SEM, ^*∗*^*p* < 0.05 (Student's* t*-test).

Statistical analysis of the PK parameters revealed that, irrespective of the administered dose, systemic exposure to the antibody was dependent on the pregnancy status. Thus, pregnant animals had a statistically significant lower AUC compared to nonpregnant controls (Figure 1). Both dose and pregnancy status contributed significantly (*p* = 0.0030 and 0.0011, resp.) to the variation in AUC values. There was no evidence of a synergistic effect between the administered dose and pregnancy status on AUC as no significant interaction between them was found (*p* = 0.7727). Additionally, pregnancy status, but not the dose, significantly affected clearance (*p* = 0.0065), mean residence time (MRT, *p* = 0.0075), and volume of distribution at steady state (*V*_ss_, *p* = 0.0171). The PK parameters that were not affected by the administered dose were pooled according to pregnancy status and reanalyzed using one-tailed Student's* t*-test (Figures 2(a)–2(d)). Only MRT remained significantly shorter in pregnant animals (*p* = 0.0274), whereas the other parameters showed nonsignificant trends towards a shorter half-life, faster clearance, and larger volume of distribution during pregnancy compared to nonpregnant controls.

Similar PK data from clinical studies during pregnancy are not readily available. In the few studies where the decrease of maternally administered IgG concentration with time has been followed, data interpretation is complicated. For example, the half-life of CMV IGIV administered to a pregnant woman with primary CMV infection was 11 days [24] compared to the 21-22 days in healthy volunteers [25], a reduction analogous to the trend in average half-life values calculated from guinea pigs (Figure 2(d)). It should be noted that the role the primary infection as well as individual differences may play in the shorter half-life in this case cannot be ruled out and should be further investigated. In another study, AUC values derived from pregnant women receiving IGIV products were not significantly different during prepregnancy period and in the first and second trimesters [26]; women in the third trimester, however, were not included in this study.

The lower AUC and higher clearance of IGIV in pregnant guinea pigs suggest that IGIV may be distributed into the fetus in pregnant animals. Indeed, in agreement with our previous studies [23] we observed a dose dependent increase of human antibody concentration in full term babies from the pregnant animals that received human IGIV product perinatally (data not shown). To ascertain that changes in maternal PK parameters correlated with placental distribution of the administered antibodies, we injected HepaGam in pregnant guinea pigs at five gestation ages roughly corresponding to end of first trimester, middle and end of second trimester, and middle and the end of the third trimester. Then we measured the IgG concentration in maternal blood and fetal blood or total body homogenates five days after injection, a time point roughly corresponding to the minimum concentration (C_min_) often termed trough level in an IGIV therapy regimen. We made the assumption that, on GD26, 35, and 45, the distribution of IgG in fetal tissues and serum is the same and made no adjustments to convert concentration in total body homogenates to fetal serum concentrations. As demonstrated by the individual data (supplemental Table 1S), the average IgG concentration in the serum of *n* = 5 GD45 litters is ~2.5-fold higher than that in whole body homogenate preparations of *n* = 2 litters from the same gestation age (Table 1S, italics). Thus, although our assumption may result in an underestimation of fetal serum concentrations, the data from GD45 indicates that the effect is of the same magnitude as individual variations in each litter.

**Figure 3:**
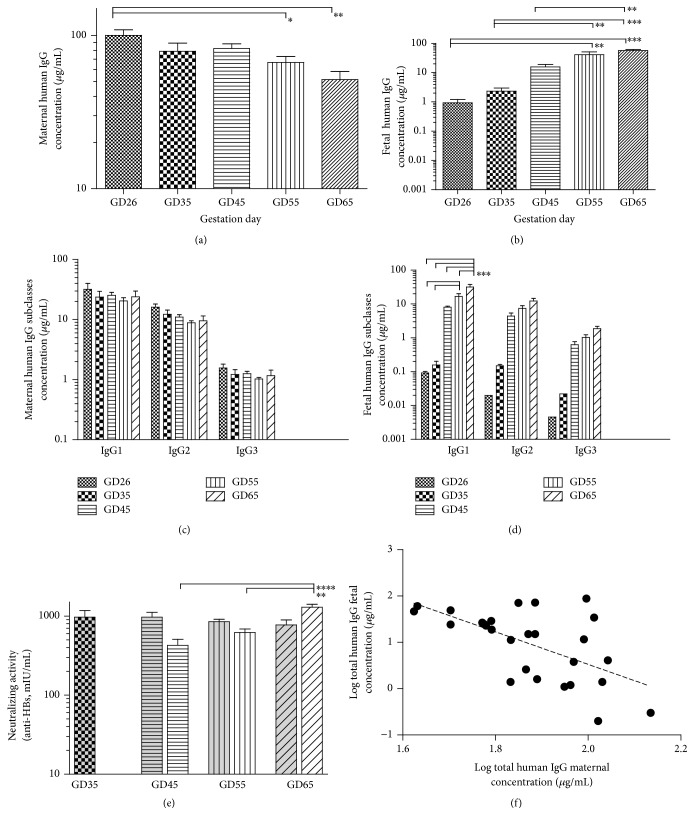
Maternal and fetal concentrations of human IgG following hepatitis B immune globulin (HBIG) administration at different gestation ages. HBIG was administered intravenously at a dose 100 IU/kg (~7 mg/kg) at different gestation ages in timed-pregnant guinea pigs and total human IgG concentrations ((a), (b), (e), and (f)), human IgG subclasses ((c), (d)), and neutralizing activity (anti-HBs, (e)) five days after administration were measured. Shown gestation days (GD, *x*-axis, or legend) roughly correspond to the end of first trimester (GD26), middle and end of second trimester (GD35 and GD45), and middle and end of third trimester (GD55 and 65). Maternal total human IgG concentrations decreased (a) and the corresponding fetal concentrations increased (b) with gestation. Similar trends were seen for human IgG subclasses in the maternal (c) and fetal (d) samples as well as the anti-HBs neutralizing activity in the mother ((e), shaded bars) and fetus ((e), clear bars). The maternal and fetal total IgG concentrations were negatively correlated (f) and, after log transformation, the relationship was linear (dotted line, Pearson *r* = −0.60, *p* = 0.0008). Data shown as mean** ± **SEM; ^*∗*^*p* < 0.05, ^*∗∗*^*p* < 0.01, ^*∗∗∗*^*p* < 0.001, and ^*∗∗∗∗*^*p* < 0.0001.

We found that the mean maternal concentrations trend progressively lower with increased gestation (Figure 3(a) and supplemental Table S1) whereas the corresponding mean fetal concentrations progressively increase (Figure 3(b), and [21]). These two variables were negatively correlated (Figure 3(f)) and, after log transformation, the relationship was linear (Pearson correlation *r* = −0.60, *p* = 0.0008, confidence interval −0.80 to −0.29). One data point from GD35 was excluded from this calculation (supplemental Table 1S, gray font and italics) due to maternal concentration being a clear outlier compared to all the other points.

While the negative correlation does not prove causality, we suggest that transplacental transfer of antibody from the mother to the baby may be a significant contributor to the decreased AUC and mean residence time we observed at the end of gestation. We further noted that not only are maternal-fetal concentration changes with increased gestation correlated, but the magnitude of changes follows the same trend. Thus, trough maternal human IgG levels are significantly lower at the middle and the end of third trimester (GD54 and 65, respectively, Figure 3(a)), the same time points where statistical significant concentration increases were seen in the fetal samples (Figure 3(b)).

We also measured concentration of all human IgG subclasses and neutralizing activity (anti-HBs levels) in both maternal and fetal samples. Given that IgG4 constitutes small percentage of HepaGam (and all other plasma derived polyclonal IgG products as well as human serum) both maternal and fetal concentrations for this subclass were below the detection limit in the majority of the collected samples. Nevertheless, we were able to detect IgG4 in some of the fetal samples in the third trimester, but most of the values were below the level of quantitation (data not shown). For the other subclasses, we observed the same general trend of decreasing maternal concentrations with increased gestation (two-way ANOVA *p* = 0.0636, Figure 3(c)), a significant increase of fetal concentrations with progression of pregnancy (two-way ANOVA *p* < 0.0001, Figure 3(d)), and a significant interaction between subclass concentrations in the fetus and gestation age (*p* = 0.0004). Post hoc analyses revealed that only IgG1 increases in GD55 and GD65 are statistically significant; the concentration increases in other subclasses do not reach significance.

We obtained similar results for the neutralizing activity (Figure 3(e)), with fetal anti-HBs levels on GD45, 55, and 65 one to two orders of magnitude higher than 10 mIU/mL, the accepted serological level of protection [27], and neutralizing antibody levels GD35 fetal blood below quantification limit.

**Figure 4:**
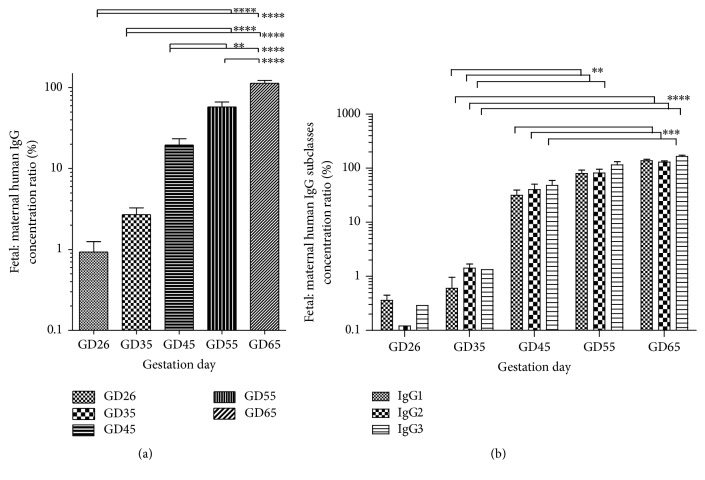
Fetal : maternal concentration ratio for total human IgG (a) and IgG subclasses (b) following hepatitis B immune globulin (HBIG) administration at different gestation ages. HBIG was administered intravenously at a dose of 100 IU/kg (~7 mg/kg) in pregnant guinea pigs at different gestation ages (GD, *x*-axis). Fetal : maternal concentration ratios (shown as mean** ± **SEM) for the total IgG and IgG subclasses 1–3 increase exponentially with gestation age. Shown time points roughly correspond to the end of first trimester (GD26), middle and end of second trimester (GD35 and 45), and middle and end of third trimester (GD55 and 65). One- and two-way ANOVA with Bonferroni post hoc analyses were used to compare total human IgG and human IgG subclasses fetal : maternal concentration ratios in each gestation age; ^*∗∗*^*p* < 0.01, ^*∗∗∗*^*p* < 0.001, and ^*∗∗∗∗*^*p* < 0.0001.

Previously we showed that pregnant guinea pigs are an appropriate animal model for studying human antibody transfer during pregnancy [21, 23]. The additional experimental data we present here enabled us to more precisely measure placental transfer and further demonstrate that this model recapitulates well the time course of the placental transfer of IgG in pregnant women. Thus, 17–22-week human fetuses have circulating concentrations of IgG that are only 5–10% of maternal levels [28] but they significantly increase during the third trimester [20, 29], often surpassing levels found in their mother. Our results show that, in the pregnant guinea pig, fetal : maternal ratios for administered human antibodies are ~3 and 20% in the middle and end of second trimester (GD35 and 45), respectively, but increase to ~60 and 110% in the middle and end of the third trimester (Figure 4(a)). Similarly to what we previously found, the fetal : maternal IgG concentration ratios with increased gestation fit an exponential growth curve (*R*^2^ = 0.87, not shown). Thus, the pharmacokinetic changes we observe in the guinea pig animal model may closely match what can be observed in women receiving IGIV during pregnancy. We should note that even though the mean fetal : maternal values for each gestation differ somewhat from what we previously calculated [21], the differences are due to an increased sample size and do not change any of the previous conclusions. All fetal : placental ratios lie within one standard deviation of previously calculated means [21] and the new group averages (Figure 4(a)) can be considered more precise point estimates of the gestation dependent placental transfer.

In a similar fashion to the total human IgG, the fetal : maternal concentration ratios for IgG subclasses 1–3 increased exponentially with gestation age (two-way ANOVA *p* < 0.0001) but, unlike the concentration changes, were similar to each other at all time points (Figure 4(b)). Post hoc analyses revealed that fetal : maternal ratios for all three IgG subclasses analyzed increased significantly in the third trimester (GD55 and 65) compared to earlier in gestation. Unlike the increases in the fetal IgG subclass concentrations (Figure 3(d)), an interaction between gestation age and subclass effects in the variance of fetal : maternal concentration ratios was not observed (*p* = 0.85).

Under our experimental conditions, we did not find any difference in placental transfer propensity for human IgG subclasses 1–3 in the pregnant guinea pig. The same has not been reported in human pregnancy [28, 30] where there is a clear difference between the fetal : maternal ratios for IgG subclasses. Some of these differences may be related to population level polymorphism in the sequence of Fc [31], but other factors have also been proposed [16]. More studies are needed to better understand the differences in placental transfer of IgG subclasses aiming to optimize the efficacy of antibody therapeutics during pregnancy.

## 4. Conclusions

Our studies in an animal model of human pregnancy show that intact human IgG molecules of all subclasses traverse the placenta at increasing levels with progression of pregnancy. This transplacental distribution can have dual implications: it may contribute to a reduction of maternal exposure to the administered antibodies compared to nonpregnant controls (Figures 1 and 3) and it can expose the fetus to progressively higher levels of therapeutic IgG with increased gestation (Figure 3). Fetal partition of the IgG, at least for HBIG and depending on the dose, may result in fetal neutralizing activity (anti-HBs levels) at time points starting with the end of second trimester (GD45) that reach and surpass the accepted serological level of protection for children and adults (Figure 3(e), [23, 27]). However, it is unknown if and at what levels neutralizing antibodies in the fetus can prevent fetal viral infections and what the effects of reduced maternal exposure to administered antibody therapy would be, especially in the presence of maternal infection. The clinical scenario may be further complicated by changes in immunity and other pregnancy related changes [7]. Thus, well-designed clinical studies and careful dosing considerations, especially in light of changes in biodistribution to the fetus at different gestation ages, are needed to assess the efficacy of therapeutic antibody treatments during pregnancy.

## Supplementary Material

The time course of human IgG concentration following intravenous administration of HepaGam® at two doses to pregnant guinea pigs 2-6 days before parturition is shown in Figure 1S. Both the average and normalized (% Cmax) IgG levels are lower in pregnant guinea pigs compared to non-pregnant controls at same time-points. Table 1S shows the individual values for maternal and fetal concentration five days following HepaGam® administration to pregnant guinea pigs at different gestation ages.

## Figures and Tables

**Table 1:** Pharmacokinetic (PK) parameters of a hepatitis B immune globulin (HBIG) product in pregnant and nonpregnant guinea pigs.

PK parameters (Mean ± SD)	3.5 mg/kg dose		7.0 mg/kg dose	
	Control (*n* = 5)	Pregnant (*n* = 5)	Control (*n* = 5)	Pregnant (*n* = 5)
AUC (*µ*g*∗*hr/mL)	8690 ± 2418	3563 ± 2500	12522 ± 3309	8100 ± 2393
CL (mL/hr per kg)	0.4 ± 0.2	1.4 ± 0.9	0.6 ± 0.2	1 ± 0.4
Half-life (hours)	107 ± 24	108 ± 40	114 ± 58	60 ± 23
MRT (hours)	53 ± 4	35 ± 15	46 ± 4	35 ± 14
*V*_ss_ (mL/kg)	23 ± 9	41 ± 11	27 ± 6	30 ± 5

AUC: area under the curve; CL: clearance; MRT: mean residence time; *V*_ss_: volume of distribution at steady state.
